# Antimicrobial action and chemical and physical properties of CuO-doped engineered cementitious composites

**DOI:** 10.1038/s41598-023-37673-1

**Published:** 2023-06-27

**Authors:** Agnieszka Ślosarczyk, Izabela Klapiszewska, Anna Parus, Sebastian Balicki, Kamil Kornaus, Bartosz Gapiński, Michał Wieczorowski, Kazimiera A. Wilk, Teofil Jesionowski, Łukasz Klapiszewski

**Affiliations:** 1 Institute of Building Engineering, Faculty of Civil and Transport Engineering, Poznan University of Technology, 60965 Poznan, Poland; 2 Institute of Chemical Technology and Engineering, Faculty of Chemical Technology, Poznan University of Technology, 60965 Poznan, Poland; 3 Department of Engineering and Technology of Chemical Processes, Faculty of Chemistry, Wroclaw University of Science and Technology, 50370 Wrocław, Poland; 4 Department of Ceramics and Refractories, Faculty of Materials Science and Ceramics, AGH University of Science and Technology, 30059 Kraków, Poland; 5 Institute of Mechanical Technology, Faculty of Mechanical Engineering, Poznan University of Technology, 60965 Poznan, Poland

**Keywords:** Chemistry, Materials science

## Abstract

CuO nanoparticles (NPs) were added to cement matrices in quantities of 0.25, 0.50 and 1.00 wt% to inhibit the growth of Gram-positive (*Bacillus cereus*, *Staphylococcus aureus*) and Gram-negative (*Pseudomonas aeruginosa*, *Escherichia coli*) bacteria. It was shown that CuO NPs, in all tested concentrations, improved the antibacterial properties of the cement matrix. Nevertheless, the best mechanical, structural and durability properties were obtained for cement composites doped with CuO NPs at 0.25 wt%. Larger amounts of NPs caused a decrease in all parameters relative to the reference mortar, which may be the result of a slight change in the porosity of the composite microstructure. For 0.50 wt% CuO NPs, a slight increase in the volume of micropores in the cement matrix was observed, and an increased number of larger pores was confirmed by non-invasive computed tomography (CT). The reduction in the mechanical parameters of composites with 0.50 and 1.00 wt% CuO NPs may also be due to the slower hydration of the cement binder, as confirmed by changes in the heat of hydration for these configurations, or agglomeration of NPs, especially for the 1.00 wt% concentration, which was manifested in a decrease in the plasticity of the mortars.

## Introduction

Literature reports in the field of civil engineering indicate that in recent years, metal nanoparticles or their oxides, or bio-based/oxide hybrid compounds, have often been proposed to achieve antibacterial and antifungal properties in building materials^1–5^. This is due to the specific mechanism of action of nanoparticles, which—unlike bulk materials—are able to penetrate the cells of microorganisms and thus inactivate them. The main role in the biocidal mechanism is played by free metal ions released from the surface of nanoparticles, and reactive oxygen species (ROS) causing oxidative stress^6,7^. It is also reported that nanoparticles with unusual shapes, in the form of rods or flowers, can interfere with the cell membrane of microorganisms, and thus start the process of their degradation^8,9^. The particles most often employed in these solutions are oxides with photocatalytic properties, such as TiO_2_^4,10–12^ and ZnO^13–15^, or metal nanoparticles. Among the latter, Ag NPs^16–18^ show the greatest ability to inhibit the multiplication and growth of both bacteria and fungi or algae. Such particles are mainly applied in the form of polymeric or silicate-based coatings, which can be incorporated for secondary protection of various surfaces of building materials, including (mainly) natural stone, concrete or wood^19–21^. Some publications indicate that the use of such coatings is much more efficient than the use of biocides, which are more toxic to the environment, and is particularly justified for buildings of historical significance^22,23^. A second way to incorporate NPs is to introduce them directly into the structure of the material by admixing them into plasters, mortars or concretes before setting. TiO_2_ and ZnO NPs have been employed most often in these solutions. Unfortunately, the biocidal effectiveness of the aforementioned NPs in building materials depends on a number of factors, the most important of which are the moisture content, access to UV radiation, surface porosity, microbial population, NP concentration, NP diameter-to-surface ratio, and the pH of the matrix^1,2^. For instance, Becerra et al*.* indicated that Ag and ZnO nanoparticles exhibit much higher antifouling activity than TiO_2_. The reason for this is the limited activity of titanium oxide, which requires UV radiation for activation^24^. On the other hand, Noeiaghaei et al*.* studied the effect of the pH of the cement matrix and the initial degradation of the cement composite with the addition of zinc oxide NPs. They showed that the stability of ZnO NPs significantly depended on the pH of the cement matrix and the tendency of the NPs to aggregate, resulting in reduced inhibition of both *B. cereus* and *E. coli* bacteria^25^.

Therefore, there is still a need to search for NPs with antimicrobial properties that will simultaneously exhibit high biocidal activity and stability in a given building material. One compound forming such NPs, as yet rarely implemented in cement-based composites, is CuO^26^. To date, copper has most often been used as a coating in the form of metallic nanoparticles alone, or as a dopant to enhance the photocatalytic activity of titanium or zinc oxide^24,27–29^. Nevertheless, reports in recent years have also indicated the effective biocidal performance of coatings with copper(II) oxide NPs alone or in combination with silica or geopolymer-based coatings^30–32^. For example, Zarzuela et al*.* showed that CuO NPs in small amounts (up to 0.35%) in combination with silica form a composite that, due to the presence of silica, reduces the porosity of the stone and delays the settling of bacteria, algae and yeast on the substrate, while the CuO nanoparticles minimize the viability of microorganisms over long periods of use^30,31^. In contrast, there are only a few publications mentioning the doping of the cement matrix with CuO NPs to achieve antibacterial properties. Sikora et al*.* pointed to the potential use of biocidal CuO nanoparticles in building materials, including cement composites^33^. However, the initial research on this topic was presented by Jędrzejczak et al*.*; they showed that CuO NPs admixed to cement mortar in a concentration of 0.50 wt% demonstrated antimicrobial effects against selected Gram-negative and positive bacteria, as well as fungi^26^.

Given the existing gap in research on the antimicrobial properties of cement composites doped with CuO NPs, this article presents comprehensive results on the effect of CuO NPs on the ability to inhibit both Gram-positive and Gram-negative bacteria, as well as on the structural, mechanical and durability properties of the cement matrix. The study also investigated the effect of copper(II) oxide on the hydration of the cement binder, and determined the porosity of the cement matrix using modern techniques, including non-invasive computed tomography.

## Experimental

### Materials

Commercially available copper(II) oxide with CAS number 1317-38-0, produced by Merck KGaA, Darmstadt, Germany, was used in the study. The copper(II) oxide used was in the form of a nanopowder with a particle size of < 50 nm (estimated by the manufacturer using TEM analysis) and a BET surface area of 29 m^2^/g. The following materials were used in the experiments on the influence of copper(II) oxide on the properties of cement composites: Portland cement CEM I 42.5 R from Górażdże Cement S.A. (Górażdże, Poland), and quartz sand (φ < 2 mm) from Kwarcmix (Tomaszów Mazowiecki, Poland).

### Characteristics of admixture

X-ray fluorescence analysis (EDXRF) was used to determine the surface composition. An Epsilon4 EDXRF spectrometer (PANalytical, Malvern, UK) was used for this test. To determine the phase composition of powdered materials, an Empyrean X-ray diffractometer (PANalytical, Malvern, UK) was used, with measurements made using monochromatic radiation. The wavelength used corresponds to the K emission line of copper, in the angular range 5–90° on a scale of 2θ, and the pitch of the goniometer was 0.008°. X’Pert HighScore Plus (version 3.0e) computer software, developed by PANalytical, was used for qualitative analysis of the phase composition. The obtained diffraction patterns were also compared with the FIZ Karlsruhe 2012 powder diffraction database and the PDF-2 database (2004). The dispersive and morphological properties of copper(II) oxide were determined by particle size measurements using a Zetasizer Nano ZS (0.6–6000 nm) instrument (Malvern Instruments Ltd., Malvern, UK) operating based on the non-invasive backscattering (NIBS) technique. To obtain information on dispersion, particle morphology and type of agglomeration in the samples, images from a VEGA 3 scanning electron microscope (Tescan Orsay Holding a.s., Brno, Czech Republic) were used.

The evaluation of electrophoretic mobility and determination of the zeta potential were based on Laser Doppler Velocimetry (LDV). Electrophoretic mobility was measured at a constant ionic strength of 0.001 M NaCl (ACS grade, CAS number: 7647-14-5, Chempur^®^, Piekary Śląskie, Poland) and the zeta potential value was then calculated based on the Henry equation. Titration was performed with a 0.2 M solution of hydrochloric acid or sodium hydroxide. Measurements were made in a pH range from 2 to 10. The mean measurement error of the zeta potential was ± 2 mV, and the measurement error of the pH value was ± 0.1.

The minimum inhibitory concentration (MIC) and minimum bactericidal concentration (MBC) were determined based on a broth microdilution method described in an article by Klapiszewska et al.^5^. The microbiological activity of CuO was evaluated against four bacterial strains: *Pseudomonas aeruginosa (G–), Escherichia coli (G–), Bacillus cereus (G*+*)* and *Staphylococcus aureus (G*+*)*. The microorganisms were first cultured for 24 h at 30 °C in sterile tryptic soy broth (TSB) culture medium, and then transferred to fresh culture medium, such that the optical density was 108 CFU/mL on the McFarland scale. Subsequently, 2 mL of an aqueous solution of resazurin (C_12_H_7_NO_4_Na—a blue redox indicator that is converted to pink resorufin in living cells) was added. Then, 50 µL of CuO suspension at the appropriate concentration was placed in a 96-well plate, and 200 µL of TSB culture medium containing resazurin indicator was added. The CuO concentrations analyzed were 0.15, 0.31, 0.62, 1.25, 2.50, 5.0, 10 and 20 mg/mL. The plates prepared in this way were incubated at 30 °C for 24 h. As control samples, culture medium with microorganisms and resazurin without CuO, as well as an abiotic sample—a solution of culture medium with resazurin and CuO in the appropriate concentration without microorganisms—were used. After incubation, the change in staining was visually assessed: pink staining in the well indicated live cells, and blue staining indicated dead cells. On this basis, MIC and MBC values were determined.

### Preparation of cement composites

A reference cement composite was produced by the standard procedure described in EN 196-1. In this procedure, 450 g of cement and 225 mL of distilled water were placed in a mixer and mixed at low speed for 60 s. In the final phase of mixing (the last 30 s), 1350 g of standard quartz sand was added. The next step was mixing at high speed (30 s), followed by a 90 s pause in mixing. After this time, mixing at high speed was resumed for another 60 s. The mortar prepared in this way was placed successively in three-part molds in two layers, with 60 strokes of a rammer being applied to compact each of them. Bars with dimensions 40 mm × 40 mm × 160 mm were removed from the molds after 24 h and stored in water until testing. The admixture of CuO in amounts of 0.25, 0.50 and 1.00 wt% was introduced into the mixer bowl in the form of a suspension in the entire volume of mixing water produced on a magnetic stirrer.

### Characteristics of cement composites

#### Flow-table test

To determine the consistency of the produced cement mortars, the flow-table test, using a shaking table, was employed. The standard test was carried out in accordance with the procedure described in EN 1015-3: the fresh mortar was placed in a wet ring on the wetted surface of the shaking table in two layers, and each layer was compacted 10 times with a rammer. In the next step, the surface was leveled, the ring was lifted vertically, and the mortar sample was subjected to 15 shocks caused by crank rotation at a speed of 1 rev/sec. The final stage was the measurement of two perpendicular diameters of the resulting mortar cake.

#### Heat of hydration

The heat of hydration test was performed using a semi-adiabatic calorimeter (Testing, Berlin, Germany) according to the procedure described in EN 196-9. The test calorimeter consisted of a thermos vessel with a thermally insulating closure and a stable aluminum enclosure that served as a mount. The mortar containers used were water–vapor-proof, and the cover (lid) was provided with a cylindrical thermometer fitting in its center. First, a fresh mortar was prepared, consisting of 360 ± 0.5 g of cement, 1080 ± 1 g of standard quartz sand and 180 ± 0.5 g of deionized water, so that the mass of the tested sample was 1575 ± 1 g. The test consisted in recording temperature changes inside the calorimeter (reference calorimeter) produced by the thermal effect. The recording of thermal parameters took place at preset time intervals, not shorter than 41 h.

#### Mechanical properties

The mechanical properties of the reference mortar and mortars doped with copper(II) oxide were tested after 7 and 28 days of maturing using the Matest Servoplus Evolution strength machine (MATEST S.p.A., Treviolo, Italy). The cement composites were removed from water, dried, weighed, and then subjected initially to a flexural strength test. In this test, the bar was placed on two support rollers and the load was carried by the third roller (the loading roller). A steady increase in force (at a rate of 0.05 kN/s) was then applied until the bar was destroyed. The two halves of the beam remaining after the test then underwent a compressive strength test. In this case, half of the bar was placed between two 40 mm × 40 mm compression plates, and then a force was applied evenly (at a rate of 2.40 kN/s) until the sample was destroyed.

#### Microstructure evaluation

Microstructural analysis of both the pure admixture and the cement composites produced was carried out using SEM images taken with a Tescan VEGA3 scanning electron microscope (Tescan Orsay Holding a.s., Brno, Czech Republic).

#### Non-invasive computed tomography

Measurements of the cement composite specimens were taken on a micro CT device. A Waygate Technologies Phoenix v|tome|x s240 computed tomograph (Hürth, Germany) was used for the study. The device was equipped with two X-ray sources—a nano and a micro tube. The nanofocus source was of the directional type and provided magnifications of up to 200x, while the microfocus tube was of the reflection target type and allowed large objects to be scanned due to an applied voltage up to 240 kV and a power of up to 320 W. A temperature-stabilized digital detector array with a pixel size of 200 µm, 1000 × 1000 pixels, with the additional option of doubling its virtual magnification, was used to obtain the image.

For this study, we employed a microfocus tube with voltage 120 kV and current 80 A. The data acquisition time for one image was 500 s. We applied the image enhancement procedure of averaging five consecutive repetitions of the image and reducing ring artifacts using the detector shifting option. The geometric magnification resulting from the position of the element between the lamp and the detector gave a magnification of 21x, which translated into a voxel size of 9.5 µm. This allowed the internal structure of the concrete samples to be imaged.

After measurement, reconstruction was performed using dedicated DATOS software. The volumetric data obtained were analyzed using Volume Graphics software. This allowed the boundary between the background and the material to be determined according to the ISO 50% rule. The porosity of the samples was then determined and the geometric dimensions—diameter and volume—were ascertained for the individual air bubbles. This enabled the non-destructive evaluation of the internal structure of samples with different additive contents.

#### Mercury porosimetry

Measurements were carried out on a Quantachrome PoreMaster 33 (Quantachrome GmbH & Co. KG, Odelzhausen, Germany). Samples were degassed at room temperature to a pressure of 10 umHg. The range of pressures during measurement was from 3 to 33,000 PSI, corresponding to pore sizes from 1 µm to 7 nm.

#### Microbial purity

Two methods were applied to determine the microbial purity of CuO-based cement composites: (*i*) a contact method on agar plates; and (*ii*) measurement of the change in optical density at 600 nm (OD600). Evaluation of microbial purity by the contact method was performed according to the procedure described in earlier papers^5,9^. The second method involved placing a 500 mg sample in a sterile 100 mL flask and adding 25 mL of sterile TSB culture medium. Then 200 µL of culture medium was taken and the optical density was measured at 600 nm. The flasks were then secured from the top to prevent contamination, placed in an incubator, and shaken for 24 h at 120 rpm. After 24 h, the culture medium was collected and the optical density at 600 nm was again measured. OD measurements were performed on a Synergy HTX spectrophotometer multi-mode reader (BioTek). Antimicrobial activity and microbial purity data are presented as mean ± SD from three identical experiments performed as three replicates.

#### Freeze–thaw test

The freeze–thaw test was used to assess durability, taking into account the degree of both internal and external damage. The test using freezing–thawing cycles was carried out in accordance with the PN-B-06265:2022 standard, in a Toropol K-012 freezing chamber with forced air circulation (ToRoPoL Sp. z o.o., Warsaw, Poland). Twelve samples of water-saturated cement mortars, after 28 days of curing, were tested. Six samples were subjected to freeze–thaw cycles, and the remaining six comparative samples were left in water for strength testing. Each period of freezing of samples (at − 18 ± 2 °C) was at least 4 h, after which the samples were thawed by complete immersion in water at a temperature of 18 ± 2 °C. The defrosting time was not less than 2 h and not more than 4 h. The samples were weighed before and after the freeze–thaw cycles, and the average weight loss of the samples after the test served as part of the evaluation and qualification criteria. For a correctly determined degree of freeze–thaw resistance, it was necessary that: *(i)* the tested samples did not show cracks, *(ii)* the total mass loss did not exceed 5% of the mass of the samples before the test, *(iii)* the decrease in compressive strength, in relation to the comparative samples, was not greater than 20%.

### Response surface methodology by D-optimal experimental design

Response surface methodology (RSM) was applied to discover the optimal doping quantity of CuO in order to fabricate cementitious composites with the potential to exhibit enhanced antibacterial performance. Furthermore, the optimal composites obtained were required to maintain satisfactory levels of physical properties such as compressive strength, porosity, and plasticity; that is, similar to or exceeding those of the reference sample. The optimization process was carried out with the assistance of a randomized quadratic D-optimal design in coordinate exchange mode, a subset of an RSM study. Design Expert Software (ver. 13.05.0) from State-Ease, Inc. (Minneapolis, USA) was used to perform all of the calculations and analysis of the results^34–36^.

To investigate the resulting response surfaces, a modified 4^1^ full quadratic D-optimal design (described in Table S1; see Supplementary Materials) was adopted, to determine the influence of the CuO doping concentration on the functional properties of the cement composites, as well as the optimal level of that concentration. The CuO concentration (A) was considered as a four-level independent variable (1—0 wt%; 2—0.25 wt%; 3—0.50 wt%; 4—1.00 wt% of CuO).

In this study, four-candidate experiments (including a reference cement sample, without added CuO; see Tables 1 and 2) enabled the formulation of a 17-run D-optimal experimental matrix—the most reliable method for implementing the experimental response factor minimization criterion. RSM assessment was used to determine the impact of the independent variable on the response factors (dependent variables). Key physical characteristics and the antibacterial functionality of the cementitious composites were recognized as response factors, as follows: Y_1_—compressive strength, Y_2_—microbial purity (CM), Y_3_—microbial purity (OD), Y_4_—porosity (MP), Y_5_—porosity (CT), Y_6_—plasticity. The following multiple linear regression equation expresses the relationship between the response factors and the independent variable based on the optimization design model^34^:

1 $${\text{Y}}_{{1}} {-}{\text{Y}}_{{6}} = \beta_{0} + \upbeta_{{1}} {\text{A }} + \upbeta_{{{1},{1}}} {\text{A}}^{{2}}$$

where Y_1_–Y_6_ are the response factors, A is the independent variable, β_0_ is an intercept term, β_1_ is the linear coefficient, and β_1,1_ is the quadratic coefficient.

**Table 1:** Oxide composition of obtained cement composites (in wt%).

	MgO	Al_2_O_3_	SiO_2_	SO_3_	K_2_O	CaO	TiO_2_	MnO	Fe_2_O_3_	CuO	Ag_2_O
CEM I	0.6	4.3	58.6	2.0	0.8	30.9	0.2	0.1	2.4	0.0	0.1
0.25 wt% CuO	0.6	4.4	58.3	2.0	0.8	31.0	0.2	0.1	2.4	0.1	0.1
0.50 wt% CuO	0.6	4.1	58.1	2.0	0.8	31.4	0.2	0.1	2.4	0.2	0.1
1.00 wt% CuO	0.6	4.4	58.3	2.0	0.8	30.7	0.2	0.1	2.4	0.4	0.1

**Table 2:** Microbiological purity and digital images obtained after 24 h by two methods, for a reference sample and composites doped with 0.25, 0.50 and 1.00 wt% CuO.

Sample	Microbial purity			
	Contact method			OD measurement
	After 24 h			
	Replicate			Replicate
				Av ± SD
Blind test	I	II	III	0.083 ± 0.001
Reference	−	+	+	0.210 ± 0.002
0.25 wt% CuO	−	−	−	0.086 ± 0.002
0.50 wt% CuO	−	−	+	0.103 ± 0.001
1.00 wt% CuO	−	−	+	0.084 ± 0.001
			
Reference	0.25 wt% CuO		0.50 wt% CuO	1.00 wt% CuO

The previously described regression models were evaluated using analysis of variance (ANOVA) and the necessary statistical parameters, *p*- and F-values. The R^2^ coefficients were used to evaluate how accurately the optimization design model fitted the experimental data. In addition, the generated multiple linear regression equations for response variables Y_1_–Y_6_ were displayed as 2D interaction effect plots, i.e., response surfaces, in order to establish the most satisfactory CuO doping concentration, leading to enhanced antimicrobial activity and appropriate physical properties. This investigation enabled determination of the correlation between the independent and dependent variables.

## Results and discussion

### Characteristics of admixture

**Figure 1:**
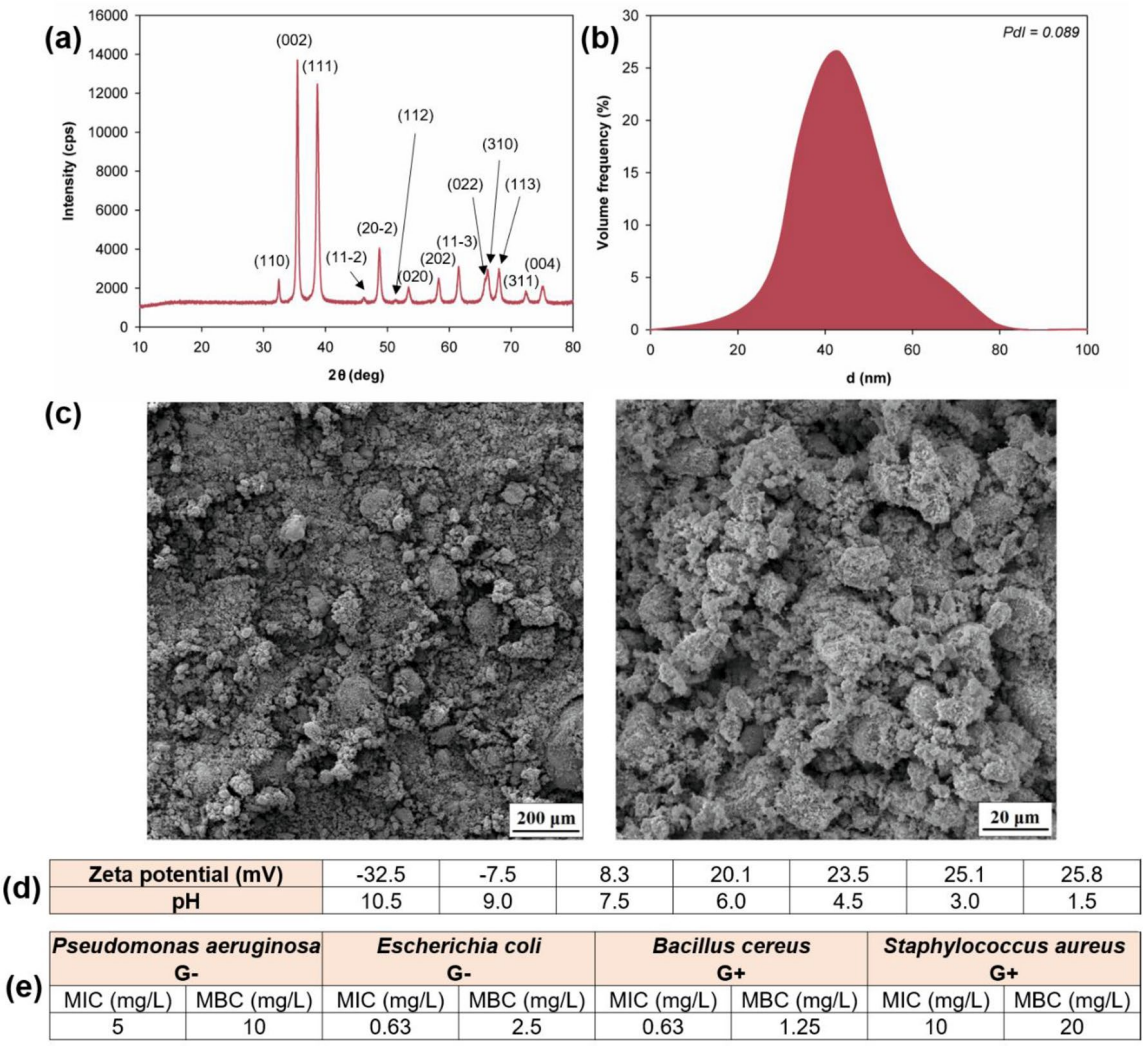
Characteristics of the copper(II) oxide: (**a**) XRD pattern, (**b**) dispersive properties, (**c**) SEM images, (**d**) zeta potential, and (**e**) antimicrobial activity.

To characterize the copper(II) oxide, XRF and XRD analyses were performed, as presented in Fig. 1a. To determine the microstructural and dispersive properties of the copper(II) oxide, the particle size distribution was measured, the polydispersity index (PdI) was determined, and SEM micrographs were analyzed. The results of these analyses are presented in Fig. 1.

The XRF analysis showed that the CuO used was 99.9% pure. The XRD spectrum for zinc oxide (see Fig. 1a) confirms the good agreement of the diffraction peaks with the standard diffraction data for CuO (JCPDS 45-0937), while no characteristic peaks were observed for other copper oxides (such as Cu_2_O or Cu_2_O_3_). Similar results were obtained by Zarei et al*.* and Mikami et al*.*^37,38^. On the basis of the data obtained, it was concluded that the CuO sample was characterized by high homogeneity (PdI = 0.089) and that the particle size of the analyzed product was in the range 24–79 nm (see Fig. 1b). This fact is confirmed by the SEM images, which clearly reveal single primary particles showing a limited tendency to aggregate and agglomerate (see Fig. 1c).

The analyzed CuO sample exhibited relatively good electrokinetic stability (see data in Fig. 1d; zeta potential greater than or equal to + 20 mV or less than or equal to − 20 mV) mainly in an alkaline environment. It should be noted that the high electrokinetic stability of samples at pH above 9 is important when this oxide is used as an admixture for cement composites, because fresh cement mortars are highly alkaline. The analyzed copper(II) oxide was also found to have an isoelectric point (IEP; a pH value at which the particles have zero electrokinetic potential). The isoelectric point occurred at pH ~ 8.5.

The analyses performed clearly indicate that CuO has high antimicrobial activity and is species-specific (see data in Fig. 1e). Based on the determined MIC and MBC values, the analyzed bacterial strains were ordered by sensitivity to CuO as follows: *Bacillus cereus* > *Escherichia coli* > *Pseudomonas aeruginosa* > *Staphylococcus aureus*. Overall, *Bacillus cereus* was the most susceptible (MIC 0.63 mg/mL, MBC 1.25 mg/mL), while *Staphylococcus aureus* showed the highest resistance among the tested microbial species (MIC around 10 mg/mL, and MBC 20 mg/mL).

There are numerous works describing the antibacterial activity of CuO, analyzing the effect of, among other things, the particle size, morphology, and the dissolution of copper ions in different media^39–41^. The microbial activity of CuO is reported to be related to such factors as the small size of NPs, the release of Cu ions, and the attraction of surface charges^42,43^. For example, Chen et al*.*^39^ described the antibacterial activity of copper oxide NPs against Gram-positive bacteria such as *Bacillus subtilis* and *Staphylococcus aureus,* as well as Gram-negative bacteria such as *Pseudomonas aeruginosa* and *Escherichia coli*, which is in accordance with the results obtained in our study. The antibacterial effect of CuO was found to be associated with a sudden decrease in cell membrane integrity and the production of reactive oxygen species.

### Characteristics of cement composites

#### Flow-table test

**Figure 2:**
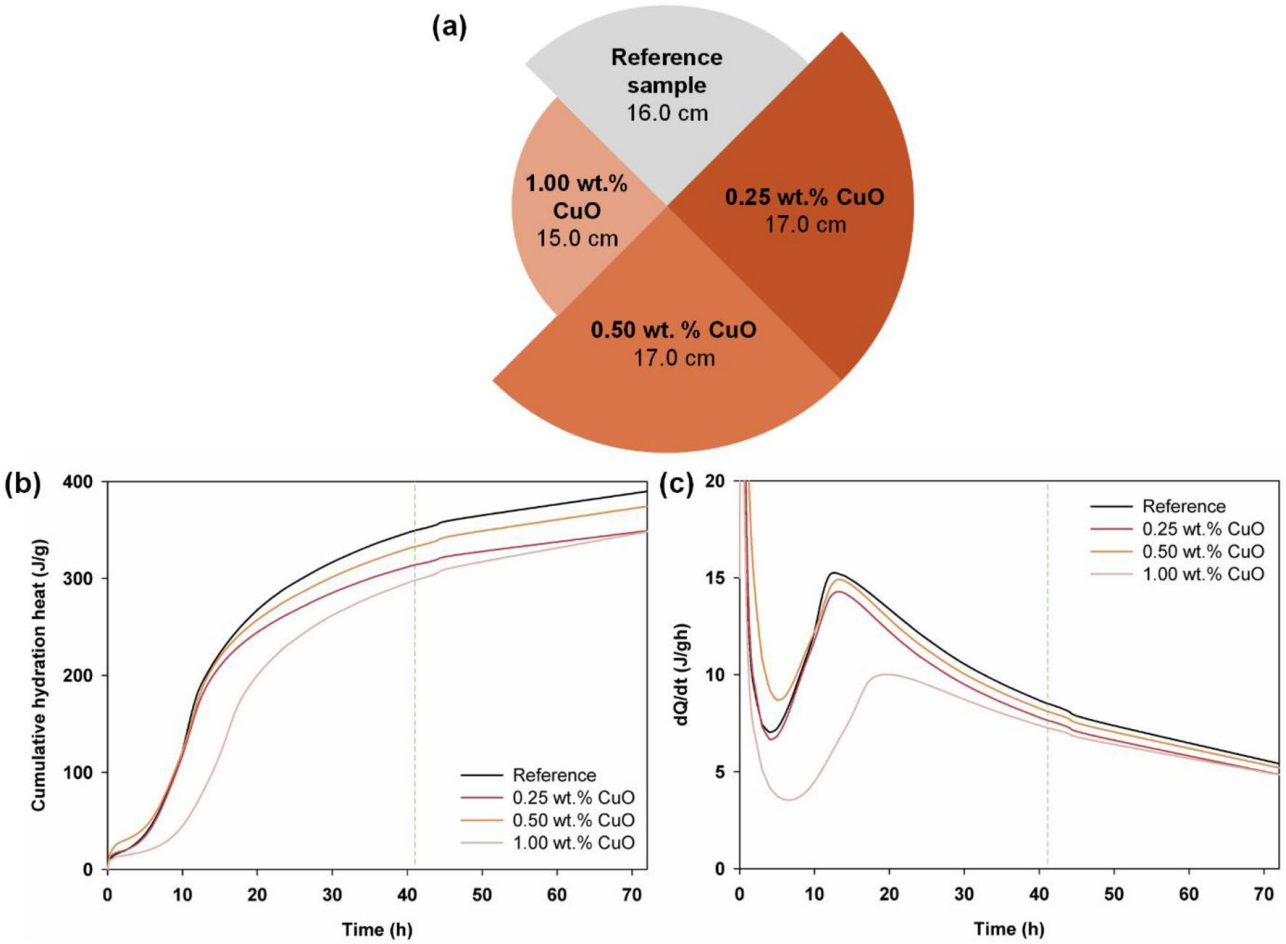
(**a**) Flow test results and (**b**,**c**) heat of hydration curves determined for the reference sample and cement composites doped with CuO, presented as the amount of heat released and the rate of heat release, respectively.

The plasticity of the cement composites was evaluated by means of a flow-table test, the results of which are shown in Fig. 2a. For the reference sample, a flow size of 16.0 cm was achieved. Samples doped with copper(II) oxide at 0.25 wt% and 0.50 wt% achieved a spread of 17.0 cm, which is 6% greater than that of the reference sample. The cement composite with a 1.00 wt% admixture of CuO exhibited the smallest flow—15.0 cm, which is 6% less than the result obtained for the reference sample. A similar study by Jędrzejczak et al*.*^26^ investigated the effect of zinc oxide (0.1 and 0.3 wt%), titanium(IV) oxide (1.0 and 2.0 wt%), 0.5 wt% copper(II) oxide, and interoxide systems on the plastic, mechanical and antibacterial properties of cement. They found that a 0.5 wt% copper(II) oxide admixture led to a spread of 16.9 cm, which corresponds to the result obtained in the present study.

#### Heat of hydration

To determine the influence of the admixtures on the thermal effect during the setting of the cement composite, a test of heat of hydration was carried out, the results of which are shown in Fig. 2b,c. The heat release curve for the cement composite without the admixture of the oxide shows a value of 349 J/g after 41 h of cross-linking, which is the highest value obtained at that time point. A value of 333 J/g (~ 5% lower) was recorded for the mortar with an admixture of 0.50 wt% CuO, and a 10% lower value of heat released was obtained for the mortar with 0.25 wt% CuO. The lowest value of the heat of hydration—more than 15% below the reference value—was obtained for the cement composite doped with 1.00 wt% CuO. The curve showing heat released for the mortar with the largest admixture of CuO presents the lowest heat of hydration in almost the entire tested range. The curve showing the rate of heat release indicates that the release of heat from this mortar occurs later than in the case of the other analyzed composites (see Fig. 2c). The reference sample and the composites with 0.25 and 0.50 wt% CuO admixtures exhibit similar heat release dynamics.

In a similar previous study^44^ in which the heat of hydration was measured using semi-adiabatic and isothermal methods for mortars and pastes containing various types and amounts of mineral admixtures (ground granulated blast furnace slag or siliceous fly ash), the results for a reference sample of CEM I 42.5R cement showed the amount of released heat to be 350 J/g, which corresponds to the heat of hydration obtained in the present study (349 J/g).

There are several literature reports in which a cement composite doped with copper(II) oxide (on a Portland cement matrix) was analyzed and the effect of this admixture on the heat of hydration released during mortar crosslinking was tested by the semi-adiabatic method. For example, in work by Zhang et al*.*^45^, the heat of hydration was measured using the isothermal method for cement pastes made of high-ferrite cement. Here, the effect of CuO in amounts of 0.2%, 0.5%, 1.0%, 1.5%, 2.0% and 3.0% was tested. The results showed that the cement paste containing 0.2% CuO released a greater amount of heat than the undoped sample, and the heat release curve for the paste with a 0.5% CuO admixture also showed a slight delay in hydration. In the case of pastes with admixtures of CuO from 1.0% upwards, lower values of released heat of hydration and delayed heat release dynamics were observed. Despite frequent differences between the two techniques for measuring the heat of hydration (isothermal and semi-adiabatic) presented by Zhang et al., the results indicate a trend and the way in which the admixture interacts with the cement matrix, which is also confirmed by the results obtained in the present work.

### X-ray fluorescence analysis

The results of energy dispersive X-ray fluorescence analysis confirm that all analyzed oxides in the reference sample correspond to components of the Portland cement composite. Importantly, the higher the content of the CuO admixture in the produced cement composites, the higher the mass percentage of copper oxide in the sample (see Table 1).

### X-ray diffraction analysis

**Figure 3:**
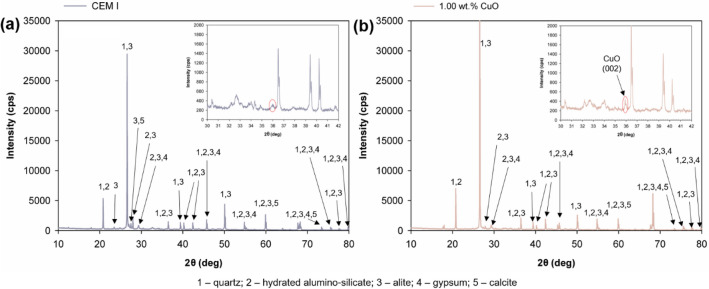
XRD patterns of (**a**) reference sample and (**b**) cement composite doped with 1.0 wt% CuO.

The results of X-ray diffraction analysis for cement composites (CEM I and 1.00 wt% CuO) are presented in Fig. 3. The numbers in the spectrum indicate the characteristic values associated with the presence of typical compounds in the cement composite matrix. These are respectively: quartz (1), tricalcium silicates and calcium aluminosilicates (2—calcium aluminum oxide silicate and 3—calcium silicate), semi-hydrated gypsum (4), and calcite (5 – calcium carbonate)^46,47^. In the spectrum of the composite with a 1.00 wt% CuO admixture (Fig. 3b), a more intense peak is visible at the 2θ value of 36°, which may indicate the presence of CuO (002). Other characteristic peaks of CuO are less visible, presumably due to their obscuring by cement matrix peaks or their low intensity (below the detection threshold).

#### Mechanical properties

**Figure 4:**
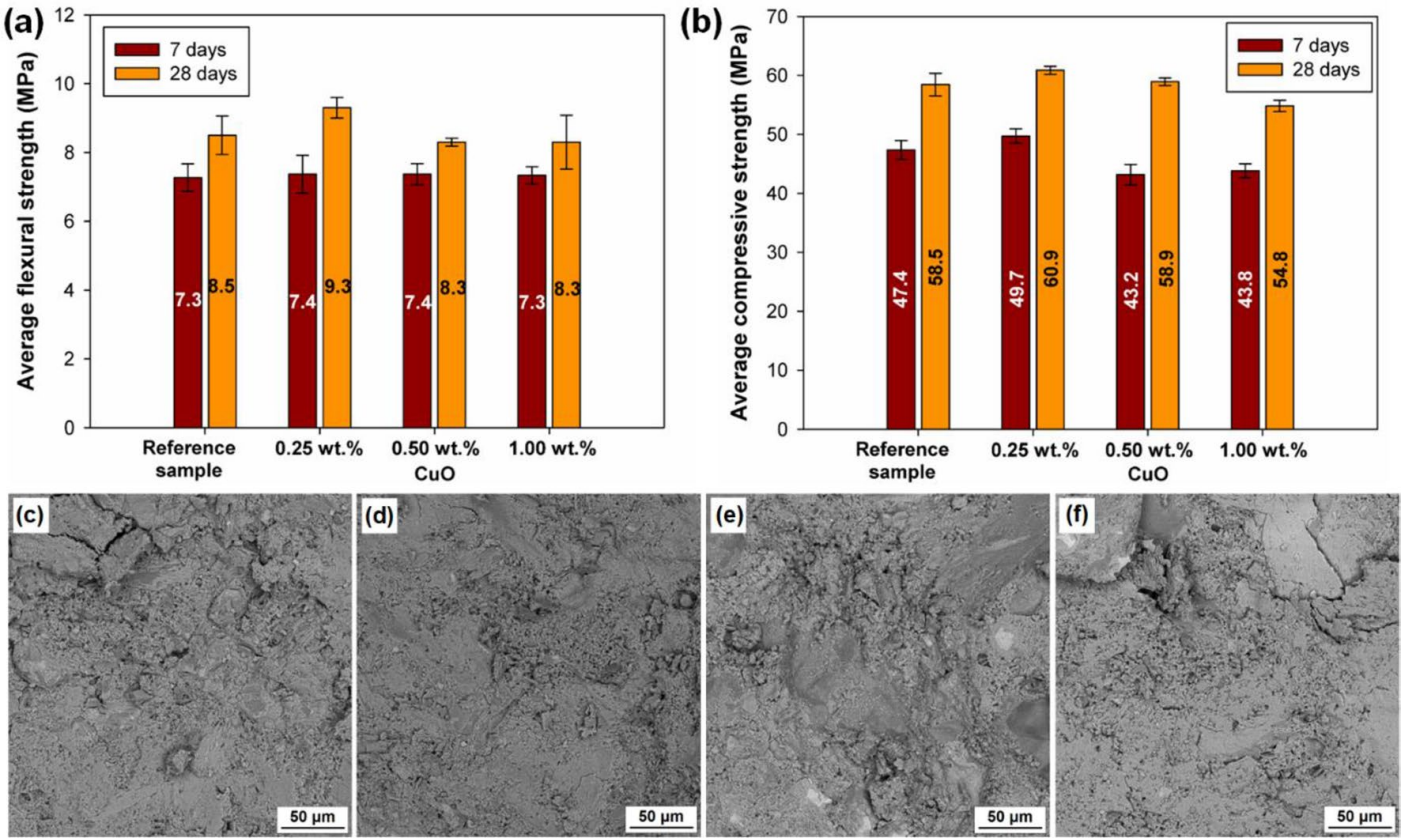
(**a**) Flexural strength and (**b**) compressive strength after 7 and 28 days of curing for the analyzed cement composites, and SEM images of (**c**) reference sample and cement composites doped with CuO at (**d**) 0.25 wt%, (**e**) 0.50 wt% and (**f**) 1.00 wt%.

The results of testing of the mechanical properties of the tested cement composites after 7 and 28 days of maturing are shown in Fig. 4a,b. The average flexural strengths (see Fig. 4a) of the reference composite are 7.3 MPa and 8.5 MPa after 7 and 28 days of maturing, respectively. In the case of composites doped with CuO, the values of the average flexural strength are very similar after 7 days of curing, regardless of the amount of the admixture. The difference in the average flexural strength between the doped samples and the reference sample becomes more significant after 28 days of curing, with the highest value achieved by the 0.25 wt% CuO sample (9.3 MPa). When the amount of CuO was increased to 0.50 and 1.00 wt%, lower flexural strengths (8.3 MPa) were obtained. These values are slightly lower (by 2.4%) than the result for the reference sample. Similar observations were made when analyzing the results for average compressive strengths (see Fig. 4b). The 0.25 wt% CuO sample achieved the highest average compressive strength (60.9 MPa) out of all tested samples, while the sample containing 0.50 wt% CuO admixture obtained a strength of 58.9 MPa, very close to that of the reference sample (58.5 MPa). The lowest average compressive strength was recorded for the sample with 1.00 wt% CuO admixture (54.8 MPa, lower than the value for the reference sample by 6.3%).

The mechanical properties of cement composites were also assessed in previous studies^45,48,49^. Ma et al*.*^48^ carried out tests on cement pastes doped with CuO in amounts of 0.5%, 1.0%, 1.5%, 2.0%, 2.5%, and 3.0%. They found that the 0.5% CuO sample had better parameters than the reference sample, but with an increase in the content of CuO, the compressive strength decreased. Similar conclusions were drawn by Zhang et al*.*^45^. They studied the effect of 0.2%, 0.5%, 1.0%, 1.5%, 2.0% and 3.0% CuO admixtures on cement pastes, and recorded the highest compressive strength for a sample with 0.2% CuO; a further increase in the proportion of CuO caused the compressive strength to decrease. Also, a study by Deng et al*.*^49^ on the influence of CuO on the compressive strength of cement pastes confirmed the trend of decreasing strength with an increase in the CuO content in the composite. Mechanical tests of copper oxide-modified cement composites show that, unlike nanosilica, copper oxide is an inert nano-additive that does not react with the cement binder^50–53^. Its action is only to densify the transition zone between the cement slurry and aggregate and to create additional active sites for the growth of the C-S-H phase. This, in turn, leads to improved strength of the composite. Similar findings have been made for other inert nano-oxides, as presented in a number of studies^50,54–59^. In addition, in the XRD analysis shown in Fig. 3 for the composite with nano-oxide, only the peak corresponding to pure copper oxide is clearly visible; no other compounds indicating the interaction of copper oxide with the cement binder phases are observed. The decrease in strength for cement composites modified with 0.50 and 1.00 wt% copper oxide indicates a slightly retarding effect of cement binder hydration. This is also indicated by the heat of hydration curves shown in Fig. 2, where lower values were observed at higher copper oxide percentages, indicating a delay in cement binder hydration relative to the reference sample. Similar trends have been observed by other researchers^48,51,60–62^.

#### Microstructure evaluation

The SEM images presented in Fig. 4c–f were used to assess the microstructure. Comparison of the microstructure of the cement composite without admixture (Fig. 4c) and composites doped with CuO (Fig. 4d–f) showed no significant changes. The structure is homogeneous, free of visible air bubbles, and well compacted. The contact zone between the aggregate and the cement binder is also very good. Similar observations were made by Jędrzejczak et al*.*^26^; they showed that the structure of a cement composite containing an admixture of 0.5 wt% CuO was dense and compact. The compactness of the structure of a cement composite containing an admixture of copper(II) oxide was also reported by Yu et al*.*^63^.

#### Non-invasive computed tomography

Non-invasive computed tomography is a method rarely used to assess the porosity of cement composites, but it makes it possible to visualize the analyzed sample, and thus to evaluate not only the number and size of pores, but also their distribution in the sample.

**Figure 5:**
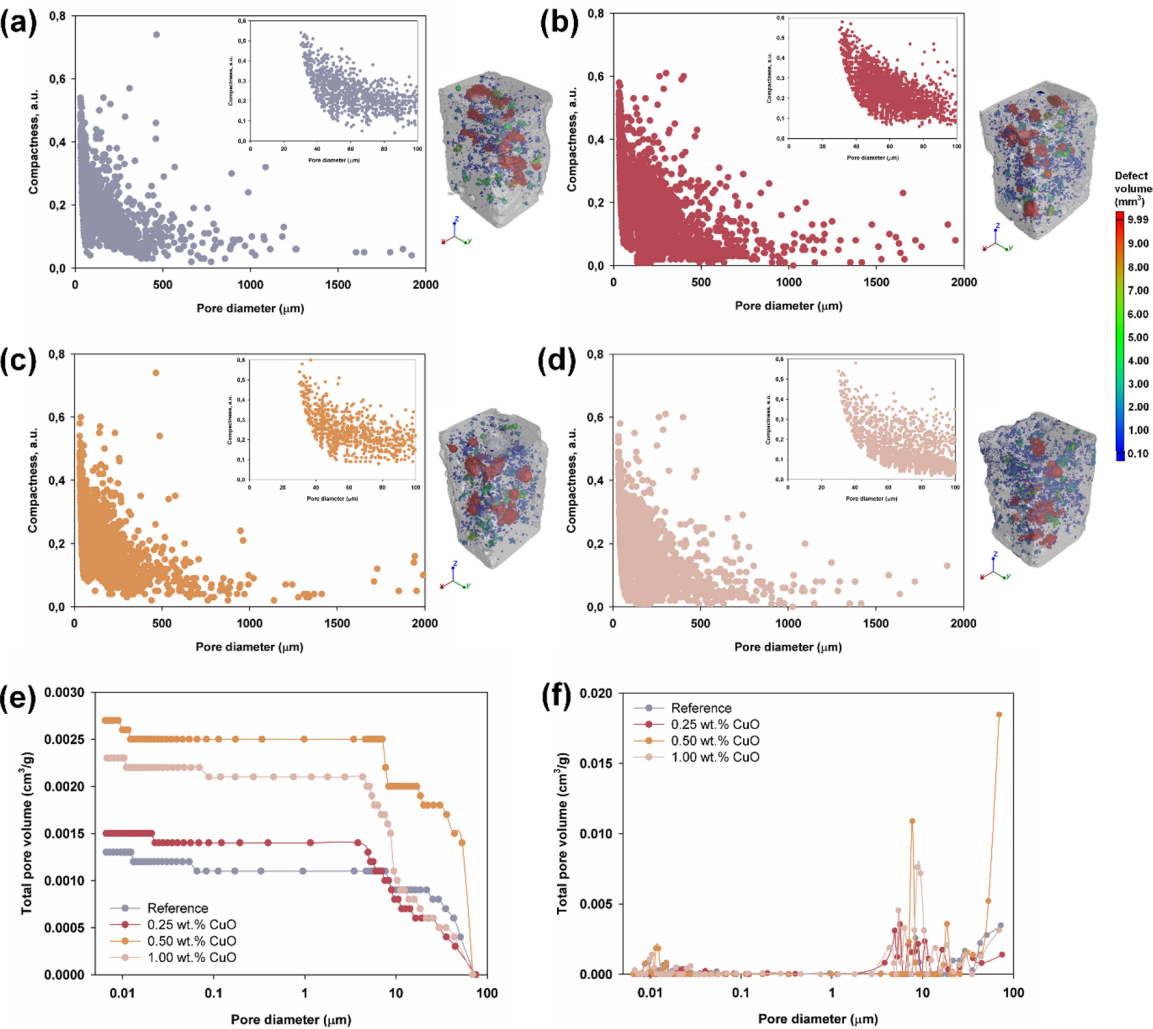
Images of samples showing the pore distributions obtained by non-invasive computed tomography (in terms of pore diameter) for composites (**a**) without admixture and with (**b**) 0.25 wt%, (**c**) 0.50 wt% and (**d**) 1.00 wt% admixtures of CuO. Additionally (**e**) cumulative curves of pore size distribution of cement mortars and (**f**) pore size distribution for the corresponding samples obtained by mercury porosimetry.

The test results obtained using this measurement technique for all analyzed cement composites are presented in Fig. 5a–d. All of the composites exhibited a compact structure with low porosity. The reference sample has the smallest pore volume. The total pore volume in the tested fragment is 5.77 mm^3^, as confirmed by the graph (see Fig. 5a). Comparison of the cement composites doped with CuO (see Fig. 5b–d) shows that the composites containing 0.25 and 1.00 wt% CuO have very similar porosities. Their total pore volumes are 6.43 and 6.34 mm^3^, respectively, compared with 7.38 mm^3^ for the composite doped with 0.50 wt% CuO. The slightly increased total pore volume for the 0.50 wt% CuO sample may be due to the higher number of larger pores in the sample. In the case of the composite containing 1.00 wt% of the admixture, a significant increase in the number of fine pores in the entire volume of the sample can be observed.

The presented visualizations of the cement composites reflect the data presented in the graphs, while also showing that both smaller and larger pores are quite evenly distributed throughout the volume of the analyzed samples.

There are few literature reports on the use of non-invasive computed tomography in the analysis of the microstructure of cement mortars. The works available mainly concern the application of X-ray microtomography^64–67^, which makes it possible to observe the structure of the cement paste, for example during hydration processes. However, the authors of these works agree that there are no other experimental techniques that would enable visualization of the entire microstructure in 3D in a non-invasive way, including the distribution, connections and nature of the pores present in the composite.

#### Mercury porosimetry

For a more accurate assessment of the porous structure, the same samples of cement composites previously tested by non-invasive computed tomography were subjected to mercury porosimetry tests, the results of which are shown in Fig. 5e,f.

The pore size distribution curves obtained for the cement mortars confirm the high compactness and density of the structure of the tested composites. The sample with the smallest total pore volume in the range < 0.01–10 μm is the reference sample. Samples doped with CuO in amounts of 0.25 and 1.00 wt% (for pores above 10 μm) are characterized by slightly smaller pore volumes. Among the tested samples, the composite containing 0.50 wt% CuO admixture had the largest total pore volume. It is notable that the trend shown in the pore size distribution curves (see Fig. 5e) coincides with the data obtained from non-invasive computed tomography. However, due to the limited measurement ranges of both techniques (> 20 μm for non-invasive computed tomography, < 100 μm for mercury porosimetry), these techniques overlap only over a small range.

Mercury porosimetry is a technique more commonly used by researchers to determine the porosity of cement composites. However, it has limitations and imperfections, which Diamond^68^ pointed out in a review article. He emphasized that the results obtained with this technique are subject to a measurement error related to the partial lack of access to some of the pores present in the hydrated cement material.

There are few literature reports in which both mercury porosimetry and non-invasive computed tomography were used to assess porosity. However, the authors of one study^69^ investigated mortar and cement paste samples using both techniques. They reported that the composite re-evaluated by computed microtomography exhibited more clearly visible pores and traces of mercury intrusion. An important conclusion from this research is that the introduction of mercury caused an increase in the volume of pores and their density, while reducing the number of pores determined using microtomography. The reason for this behavior may be the pressure generated during mercury injection, which in some cases can be destructive.

### Microbial purity

Samples of cements with different amounts of CuO admixture were evaluated for microbiological purity, that is, for the presence of microorganisms that can grow and colonize the material under favorable conditions. The results are shown in Table 2.

Pure cement without the addition of CuO, as in previous studies^5,9^, exhibited the presence of microbial colonies, confirming that it is susceptible to colonization by microorganisms. In contrast, in tests performed using the contact method, the microbial purity of the cement samples containing CuO was found to be high, with a few small colonies observed in one out of three replicates (see Table 2).

Analogous results were obtained by measuring the change in optical density (OD600). Cultures conducted in the presence of cement samples containing CuO show no change in optical density after 24 h incubation (see Table 2). As different concentrations of CuO were used in the study, we can conclude from the observations that the introduction of as little as 0.25 wt% CuO additive causes strong inhibition of microbial growth.

The antibacterial properties of copper(II) oxide have been the focus of several studies, as described by Omar et al*.*^70^ and Acikbas et al.^71^, among others. Their results on the microbial activity of CuO correspond well with our results, particularly the evaluation of microbial purity in which no microbial growth was observed in the cement samples with added CuO. Particularly interesting results were obtained in the study by Acikbas et al*.*^71^, in which a layer of CuO was applied to the surface of ceramic tiles. These tiles displayed high antibacterial activity against *S. aureus* and *E. coli*. The authors also indicate that the crystallization of the tenorite phase has a strong influence on the antibacterial activity.

The results obtained in this study confirm that the addition of CuO to cement is a viable method of inhibiting the growth of microorganisms. This is particularly promising from the point of view of practical application, since the introduction of CuO as an additive to cement creates the possibility of controlling the growth of certain species of microorganisms, which in turn is undoubtedly important in locations requiring a sterile environment (such as hospitals or nurseries).

### Freeze–thaw test

To assess the durability of cement composites doped with CuO, 150 freeze–thaw cycles were carried out. The results of this test are presented in Table 3.

**Table 3:** Results of freeze–thaw tests for all analyzed samples.

Sample	Mean mass loss after the test (%)	Mean mass loss after the test (g)	Average strength of samples (N/mm^2^)		Difference in endurance (%)
			Reference	Examined	
CEM I	0.96	5.8	66.0	63.3	4.0
0.25 wt% CuO	0.71	4.2	70.4	64.4	8.5
0.50 wt% CuO	1.25	7.4	69.3	62.8	9.5
1.00 wt% CuO	0.70	4.2	66.1	61.8	6.0

The data confirm the improvement in the mechanical strength of cement composites produced with a CuO admixture relative to the reference sample (see average strengths of comparative samples) after a longer period of maturation. Despite a 9% decrease in the average compressive strength, the composite doped with 0.25 wt% CuO gave the highest value of mechanical strength (64.4 MPa) among all tested composites. This sample also achieved the smallest weight loss (4 g). All of the analyzed samples met the requirements of the freeze–thaw test and obtained the level of resistance defined as F150.

A study by Dvorkin^72^ analyzed the parameters of a concrete structure that affect its frost resistance. One such factor is the porosity of the cement composite. That study also found that the w/c of the composites had a significant impact on the number of freeze–thaw tests successfully performed. Materials with potentially favorable frost resistance parameters are those with w/c < 0.7.

In agreement with the correlation shown by Dvorkin^72^ between frost resistance and the characteristics of the porous structure, it was observed that the cement composite without admixture and having the lowest pore content was the material with the smallest loss of strength after 150 freeze–thaw cycles. The cement composite doped with 0.25 wt% CuO, having a slightly higher content of fine pores and a smaller content of larger ones, despite achieving the highest average compressive strength, exhibited a decrease in strength by 8.5%. The composite with the highest porosity, the mortar with an admixture of 0.50 wt% CuO, produced the greatest decrease in the average compressive strength. The last of the tested composites, doped with 1.00 wt% CuO and having average porosity, suffered a strength loss of 6%, which (like its porosity) is an average value among all of the tested systems. It is interesting to observe that the composites with 0.25 and 1.00 wt% admixtures had higher contents of fine pores than the reference composite, but smaller contents of larger pores, which may be a reason for their lower mass losses after the completion of freezing–thawing tests.

### Optimization of CuO doping concentration in cementitious composites

RSM is frequently employed in evaluation studies, as it is one of the most effective statistical tools for various process optimizations. It can provide a useful solution to a problem when the direct influence of the independent variables (process parameters) on the dependent variables (response factors) must be determined, generally in complex systems where the variables may be correlated in various ways, which makes it desirable to establish their individual and combined effects^36,73^.

Using a randomized quadratic D-optimal design with coordinate exchange, this study examined the effect of varying percentages of CuO additive on the antimicrobial functional qualities and necessary physical properties of fabricated cement composites. The direct effect of the single independent variable of the evaluated candidates, namely the concentration of CuO doping (A), on the dependent variables—Y_1_ (compressive strength), Y_2_ (microbial purity CM), Y_3_ (microbial purity OD), Y_4_ (porosity MP), Y_5_ (porosity CT), and Y_6_ (plasticity)—was studied for statistical significance. Table S1 presents the experimental matrix of the randomized D-optimal design with 17 different experimental runs, along with the levels of the independent variable and the experimental values of the response factors for each run.

In the current work, the 2D interaction effect plots (response surfaces) graphically represent the multiple linear regression model functions deriving from the randomized quadratic D-optimal design. By investigating response surfaces, potential correlations between independent and dependent variables were evaluated. The primary objective of the optimization was to establish the best concentration of CuO additive in cement composite, which will enable the fabrication of cement samples enriched with copper(II) oxide particles and result in enhanced antibacterial properties. Tables 2, 3 show the specific cement composites, including the reference sample and composites doped with specified CuO concentrations.

**Figure 6:**
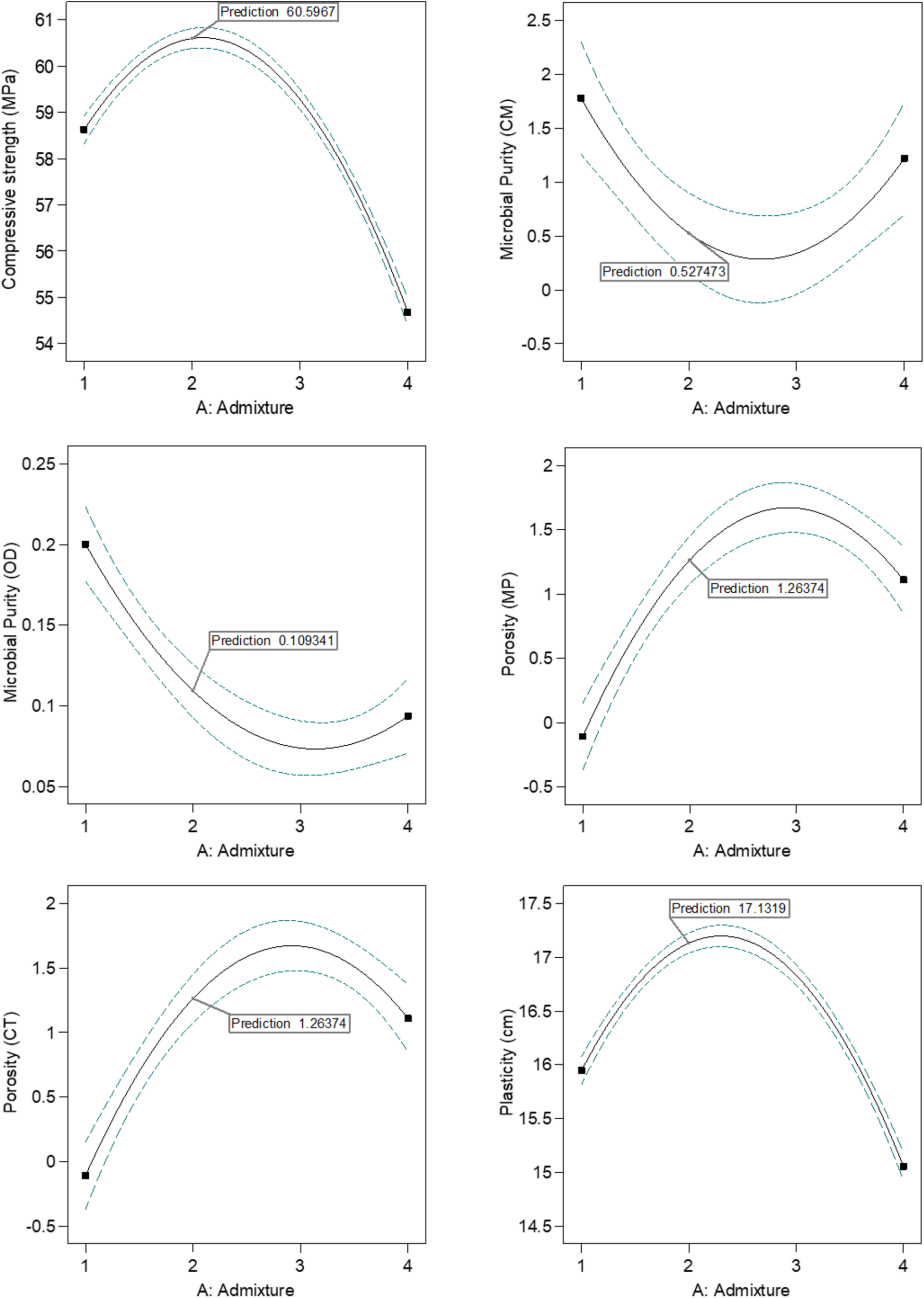
Randomized quadratic D-optimal design interaction effect plots (response surfaces) for the response factors Y_1_ (compressive strength), Y_2_ (microbial purity CM), Y_3_ (microbial purity OD), Y_4_ (porosity MP), Y_5_ (porosity CT), and Y_6_ (plasticity) vs. the single independent variable: concentration of CuO doping (A).

As shown by the general trend in the 2D response plots in Fig. 6, an increase in CuO doping concentration has a positive quadratic effect on the physical characteristics and a negative quadratic effect on the antimicrobial functionality of the studied composites, with local minima and maxima. As a result of the statistical analysis and fitting of the experimental model, the regions for the optimal concentration of CuO admixture were found to be around 0.25 percent in the 2D plots (marked by the prediction values in Fig. 6). As indicated by the design model’s optimal CuO concentration, the CuO-doped cement composites met the requirements for high microbial purity; that is, a value of purity as close to zero as possible. Moreover, the measured physical properties of the 0.25 wt% CuO samples were at desirable levels, having the highest compressive strength among all samples, elevated plasticity, and a satisfactory level of porosity. Therefore, the results of the RSM analysis are consistent with and support the results presented in the previous sections.

The mathematical and statistical modeling-based evaluation techniques of RSM and design of experiments (DoE) have been shown to be effective in predicting and verifying the functional properties of chemical formulations and the end products of optimized production processes^74,75^. Analyses of variance (ANOVA tests) of the response surfaces produced by the randomized D-optimal design for dependent variables Y_1_–Y_6_ demonstrated that the quadratic multiple linear regression model provided the best fit in all circumstances, which is a common result in optimization by RSM and DoE, as reported in the literature^36,73,76,77^. The above-mentioned best-fit model had notable statistical parameters, namely an insignificant lack of fit with an appropriate number of degrees of freedom, and strongly matched R^2^ coefficients (experimental, adjusted, and predicted). For each of the six expected response factors in this investigation, the resulting *p*-values of model fitting were less than 0.05, indicating that the D-optimal design chosen for optimization was statistically significant. Tables S1–S6 summarize the ANOVA results (see Supplementary Materials). In the case of the compressive strength response (Y_1_), there was both a linear and a strong quadratic effect of CuO concentration, with one local maximum, namely the highest exhibited strength for 0.25 wt% of CuO, which was one of the optimization goals (besides microbial activity). Moreover, increasing the amount of CuO in the mixture would have a negative effect on the compressive strength. There was also a linear and quadratic effect of the amount of CuO on the microbial purity response factors (Y_2_ and Y_3_), but the required minimum values of antimicrobial activity were reached only in the local minimal region, namely 0.25–0.50 wt% of CuO in the cement composite. Finally, for the remaining response factors Y_4_–Y_6_ (porosities and plasticity), there was a visible trend as in the case of Y_1_, with a linear and strong quadratic effect, but the local maximum region was in the range 0.25–0.50 wt% of CuO doping in the cement composite. Nevertheless, the ultimate goal of the optimization was to achieve the highest compressive strength among all cement composite samples with the lowest possible microbial purity, while maintaining the values of the remaining physical properties at satisfactory levels, and this affected the selection of the optimal candidate.

The following multiple linear regression formulae were obtained using a D-optimal model that was fitted to the observed values of the response variables, and they serve as a summary of the ANOVA tests that were run:

2 $${\text{Compressive strength}} = + 60.35 \, {-}1.98{\text{A }}{-}3.70{\text{A}}^{2}$$

3 $${\text{Microbial Purity }}\left( {{\text{CM}}} \right) \, = \, + 0.3008 \, {-}0.2802{\text{A }} + 1.20{\text{A}}{}^{2}$$

4 $${\text{Microbial Purity }}\left( {{\text{OD}}} \right) \, = \, + 0.0847 \, {-}0.0533{\text{A }} + 0.0623{\text{A}}^{2}$$

5 $${\text{Porosity }}\left( {{\text{MP}}} \right) = + 1.59 \, + 0.6099{\text{A }}{-}1.09{\text{A}}^{2}$$

6 $${\text{Porosity }}\left( {{\text{CT}}} \right) \, = \, + {1}.{59 } + 0.{6}0{\text{99A }}{-}{1}.0{\text{9A}}^{2}$$

7 $${\text{Plasticity }} = \, {-}0.0{7}0{9 }{-}{3}.{\text{37A }}{-}0.{\text{4123A}}^{2}$$

Those formulae support the analyzed relations between response factors and the single independent variable of the cementitious composites doped with CuO, resulting in the formation of cement composite samples with desired enhanced microbial purity and satisfactory physical properties. The final conclusion derived after statistical analysis and exploration of the response surfaces during optimization, expressed in the candidate profile (see Table 4), together with the desirability function, enabled selection of the best solution, namely a cementitious composite doped with 0.25 wt% CuO.

**Table 4:** Selected candidate for the optimal cementitious composite, samples doped with 0.25 wt% CuO, proposed by D-optimal model optimization, based on a desirability function, comparison of predicted and actual values, and compromise between microbial purity and physical properties.

Selected admixture	Compressive strength (MPa)	Microbial purity (CM)	Microbial purity (OD)	Porosity (MP)	Porosity (CT)	Plasticity	Desirability
2–0.25 wt% CuO							
Predicted values	60.597	0.527	0.109	1.264	1.264	17.132	0.727
Actual values	60.9	0	0.086	1	1	17.0	–

## Conclusion

As part of this research, the basic physicochemical parameters of copper(II) oxide were determined. Among these were a particle size distribution in the range 255–825 nm (PdI = 0.096) and relatively high electrokinetic stability (at pH > 9), resulting in good synergy with the alkaline cement matrix. In the study, CuO was used as an admixture in cement mortars in amounts of 0.25, 0.50 and 1.00 wt%, and an analysis was made of its influence on plasticity (plasticity increased for 0.25 and 0.50 wt% admixtures), mechanical properties after 7 and 28 days of curing (the best properties were those of the system doped with 0.25 wt% CuO), and microstructure (the CuO admixture thickens and seals the structure). Tests of heat of hydration, using the semi-adiabatic method, were also carried out, and showed slight changes in the course of the curves obtained for composites doped with CuO. An important aspect of the work was the assessment of the porosity of cement composites, based on two measurement techniques rarely used together by researchers: mercury porosimetry and non-invasive computed tomography. The results presented show a number of similarities and limitations related to the use of these research methods. Still, both confirmed the packed structure of the composites produced. Moreover, valuable information was provided by microbiological purity tests carried out on pure admixture and cement composites with CuO. The antimicrobial effect of CuO and cement composites doped with CuO (preferably 0.25 wt% CuO) was confirmed. The research also included freeze–thaw tests, which showed the durability of all tested composites to be in the F150 class.

## Supplementary Information

Supplementary Tables.

## Data Availability

All data generated or analyzed during this study are included in this published article and its supplementary information files.
