# Supplementary material for: Antimicrobial action and chemical and physical properties of CuO-doped engineered cementitious composites
Source: Sci Rep. 2023 Jun 27;13:10404. doi: 10.1038/s41598-023-37673-1 (PMC10300130; doi:10.1038/s41598-023-37673-1)
Supplement: Supplementary file 1 — Supplementary Tables. [file 41598_2023_37673_MOESM1_ESM.docx]

**Supplementary Materials**

**Production of CuO-doped engineered cementitious composites:
Antimicrobial action, chemical and physical assessment**

Agnieszka Ślosarczyk^a,^*, Izabela Klapiszewska^a^, Anna Parus^b^, Sebastian Balicki^c^,
Kamil Kornaus^d^, Bartosz Gapiński^e^, Michał Wieczorowski^e^, Kazimiera A. Wilk^c^,
Teofil Jesionowski^b^, Łukasz Klapiszewski^b,^*

^a^ *Institute of Building Engineering, Faculty of Civil and Transport Engineering,
Poznan University of Technology, PL-60965 Poznan, Poland*

^b^ *Institute of Chemical Technology and Engineering, Faculty of Chemical Technology, Poznan University of Technology, PL-60965 Poznan, Poland*

^c^ *Department of Engineering and Technology of Chemical Processes, Faculty of Chemistry, Wrocław University of Science and Technology, PL-50370 Wroclaw, Poland*

*^d^ Department of Ceramics and Refractories, Faculty of Materials Science and Ceramics, AGH University of Science and Technology, PL-30059 Kraków, Poland*

^e^ *Institute of Mechanical Technology, Faculty of Mechanical Engineering,
Poznan University of Technology, PL-60965 Poznan, Poland*

*^*^Correspondence: lukasz.klapiszewski@put.poznan.pl; Tel.: +48 61-665-37-48 (Ł.K.);*

*agnieszka.slosarczyk@put.poznan.pl; Tel.: +48 61-665-21-66 (A.Ś.)*

**Table S1.** A randomized quadratic *D*-optimal design’s experimental matrix of singular independent variable (A) with corresponding levels and analyzed response factors Y_1_ – Y_6_: compressive strength, microbial purity (CM), microbial purity (OD), porosity (MP), porosity (CT) and plasticity, respectively.

| **Run** | **Factor A:**  **admixture type^*^** | **Response Y_1_:**  **Compressive strength (MPa)** | **Response Y_2_: Microbial purity (CM)** | **Response Y_3_: Microbial purity (OD)** | **Response Y_4_: Porosity (MP)** | **Response Y_5_: Porosity (CT)** | **Response Y_6_: Plasticity (cm)** |
| --- | --- | --- | --- | --- | --- | --- | --- |
| 1 | 4 | 54.8 | 1 | 0.084 | 1 | 1 | 15 |
| 2 | 3 | 58.9 | 1 | 0.103 | 2 | 2 | 17 |
| 3 | 1 | 58.5 | 2 | 0.210 | 0 | 0 | 16 |
| 4 | 4 | 54.8 | 1 | 0.084 | 1 | 1 | 15 |
| 5 | 4 | 54.8 | 1 | 0.084 | 1 | 1 | 15 |
| 6 | 2 | 60.9 | 0 | 0.086 | 1 | 1 | 17 |
| 7 | 1 | 58.5 | 2 | 0.210 | 0 | 0 | 16 |
| 8 | 3 | 58.9 | 1 | 0.103 | 2 | 2 | 17 |
| 9 | 3 | 58.9 | 1 | 0.103 | 2 | 2 | 17 |
| 10 | 1 | 58.5 | 2 | 0.210 | 0 | 0 | 16 |
| 11 | 2 | 60.9 | 0 | 0.086 | 1 | 1 | 17 |
| 12 | 2 | 60.9 | 0 | 0.086 | 1 | 1 | 17 |
| 13 | 2 | 60.9 | 0 | 0.086 | 1 | 1 | 17 |
| 14 | 1 | 58.5 | 2 | 0.210 | 0 | 0 | 16 |
| 15 | 3 | 58.9 | 1 | 0.103 | 2 | 2 | 17 |
| 16 | 2 | 60.9 | 0 | 0.086 | 1 | 1 | 17 |
| 17 | 4 | 54.8 | 1 | 0.084 | 1 | 1 | 15 |

* 1 – CEM, 2 – 0.25 wt.% CuO, 3 – 0.5 wt.% CuO, 4 – 1.0 wt.% CuO

**Table S2.** ANOVA results for *D-*optimal randomized quadratic model for response factor Y_1_ – compressive strength.

**Response 1: Compressive strength (MPa)**

| **Source** | **Sum of Squares** | **df** | **Mean Square** | **F-value** | **p-value** |
| --- | --- | --- | --- | --- | --- |
| **Model** | 82.95 | 2 | 41.48 | 499.44 | < 0.0001 |
| A-Admixture | 35.11 | 1 | 35.11 | 422.74 | < 0.0001 |
| A² | 45.76 | 1 | 45.76 | 550.97 | < 0.0001 |
| **Residual** | 1.16 | 14 | 0.0830 |  |  |
| **Cor Total** | 84.12 | 16 |  |  |  |
| **R² =** 0.9862, **Adjusted R² =** 0.9842, **Predicted R² =** 0.9814  Compressive strength = +60.35 -1.98A -3.70A² | | | | | |

**Table S3.** ANOVA results for *D-*optimal randomized quadratic model for response factor Y_2_ – microbial purity (CM).

**Response 2: Microbial Purity (CM)**

| **Source** | **Sum of Squares** | **df** | **Mean Square** | **F-value** | **p-value** |
| --- | --- | --- | --- | --- | --- |
| **Model** | 5.42 | 2 | 2.71 | 10.80 | 0.0015 |
| A-Admixture | 0.7057 | 1 | 0.7057 | 2.81 | 0.1159 |
| A² | 4.81 | 1 | 4.81 | 19.15 | 0.0006 |
| **Residual** | 3.52 | 14 | 0.2512 |  |  |
| **Cor Total** | 8.94 | 16 |  |  |  |
| **R² =** 0.6067, **Adjusted R² =** 0.5505, **Predicted R² =** 0.4698  Microbial Purity (CM) = +0.3008 -0.2802A +1.20A² | | | | | |

**Table S4.** ANOVA results for *D-*optimal randomized quadratic model for response factor Y_2_ – microbial purity (OD).

**Response 3: Microbial Purity (OD)**

| **Source** | **Sum of Squares** | **df** | **Mean Square** | **F-value** | **p-value** |
| --- | --- | --- | --- | --- | --- |
| **Model** | 0.0376 | 2 | 0.0188 | 38.23 | < 0.0001 |
| A-Admixture | 0.0255 | 1 | 0.0255 | 51.87 | < 0.0001 |
| A² | 0.0130 | 1 | 0.0130 | 26.43 | 0.0001 |
| **Residual** | 0.0069 | 14 | 0.0005 |  |  |
| **Cor Total** | 0.0445 | 16 |  |  |  |
| **R² =** 0.8452, **Adjusted R² =** 0.8231, **Predicted R² =** 0.7914  Microbial Purity (OD) = +0.0847 -0.0533A +0.0623A² | | | | | |

**Table S5.** ANOVA results for *D-*optimal randomized quadratic model for response factor Y_2_ – porosity (MP).

**Response 4: Porosity (MP)**

| **Source** | **Sum of Squares** | | **df** | **Mean Square** | **F-value** | **p-value** |
| --- | --- | --- | --- | --- | --- | --- |
| **Model** | 7.12 | 2 | | 3.56 | 56.70 | < 0.0001 |
| A-Admixture | 3.34 | 1 | | 3.34 | 53.24 | < 0.0001 |
| A² | 3.96 | 1 | | 3.96 | 63.03 | < 0.0001 |
| **Residual** | 0.8791 | 14 | | 0.0628 |  |  |
| **Cor Total** | 8.00 | | 16 |  |  |  |
| **R² =** 0.8901, **Adjusted R² =** 0.8744, **Predicted R² =** 0.8518  Porosity (MP) = +1.59 +0.6099A -1.09A² | | | | | | |

**Table S6.** ANOVA results for *D-*optimal randomized quadratic model for response factor Y_2_ – porosity (CT).

**Response 5: Porosity (CT)**

| **Source** | **Sum of Squares** | **df** | **Mean Square** | **F-value** | **p-value** |
| --- | --- | --- | --- | --- | --- |
| **Model** | 7.12 | 2 | 3.56 | 56.70 | < 0.0001 |
| A-Admixture | 3.34 | 1 | 3.34 | 53.24 | < 0.0001 |
| A² | 3.96 | 1 | 3.96 | 63.03 | < 0.0001 |
| **Residual** | 0.8791 | 14 | 0.0628 |  |  |
| **Cor Total** | 8.00 | 16 |  |  |  |
| **R² =** 0.8901, **Adjusted R² =** 0.8744, **Predicted R² =** 0.8518  Porosity (CT) = +1.59 +0.6099A -1.09A² | | | | | |

**Table S7.** ANOVA results for *D-*optimal randomized quadratic model for response factor Y_2_ – plasticity.

**Response 6: Plasticity**

| **Source** | **Sum of Squares** | **df** | **Mean Square** | **F-value** | **p-value** |
| --- | --- | --- | --- | --- | --- |
| **Model** | 11.31 | 2 | 5.65 | 360.21 | < 0.0001 |
| A-Admixture | 1.78 | 1 | 1.78 | 113.40 | < 0.0001 |
| A² | 9.32 | 1 | 9.32 | 593.37 | < 0.0001 |
| **Residual** | 0.2198 | 14 | 0.0157 |  |  |
| **Cor Total** | 11.53 | 16 |  |  |  |
| **R² =** 0.9809, **Adjusted R² =** 0.9782, **Predicted R² =** 0.9743  Plasticity = -0.0709 -3.37A -0.4123A² | | | | | |
